# The Application of Functionalized Pillared Porous Phosphate Heterostructures for the Removal of Textile Dyes from Wastewater

**DOI:** 10.3390/ma10101111

**Published:** 2017-09-21

**Authors:** José Jiménez-Jiménez, Manuel Algarra, Vanessa Guimarães, Iuliu Bobos, Enrique Rodríguez-Castellón

**Affiliations:** 1 Departamento de Química Inorgánica, Facultad de Ciencias, Universidad de Málaga, Campus de Teatinos s/n, 29071 Málaga, Spain; jjimenez@uma.es (J.J.-J.); malgarra67@gmail.com (M.A.); 2 Instituto de Ciências da Terra, Porto, DGAOT, Faculdade de Ciências, Universidade do Porto, Rua do Campo Alegre 687, 4169-007 Porto, Portugal; guimavs@gmail.com (V.G.); ibobos@fc.up.pt (I.B.)

**Keywords:** dye remediation, adsorption, azo dye, wastewater, pillared porous phosphate heterostructures, isotherm

## Abstract

A synthesized functionalized pillared porous phosphate heterostructure (PPH), surface functionalized phenyl group, has been used to remove the dye Acid Blue 113 from wastewater. X-ray photoemission spectroscopy XPS and X-ray diffraction (XRD) were used to study its structure. The specific surface area of this was 498 m^2^/g. The adsorption capacities of PPH and phenyl surface functionalized (Φ-PPH) were 0.0400 and 0.0967 mmol/g, respectively, with a dye concentration of 10^−5^ M when well fitted with SIPS and Langmuir isotherms respectively (pH 6.5, 25 °C). The incorporation of the dye to the adsorbent material was monitored by the S content of the dye. It is suggested as an alternative for Acid Blue 113 remediation.

## 1. Introduction

Nowadays, among the concerns that must be dealt with on a global level are toxic and carcinogenic environmental pollutants. A large portion of toxic contaminants are dyes, used for dyeing textiles and other industrial purposes. Many physical, chemical and biophysical processes have been studied for their ability to remove environmental pollutants [1]. In particular, the development of new technologies for the easy decolorization or remediation of these types of compounds is of current interest.

Dyes used in the textile industry include a wide range of chemical compounds, based mainly on substituted aromatic structures linked by azo groups [2]; therefore textile dye wastewater is based not only in color removal but also in the degradation, mineralization and total removal. A large variety of techniques has been used including chemical methods such as coagulation [3,4,5], flocculation [6,7,8] or precipitation [9,10], and new photocatalytic processes such as the Fenton reaction [11,12] are being implemented and optimized; biological with aerobic and anaerobic microbial degradation [13,14,15], and the use of pure enzymes [16,17] have been investigated to overcome physical methods such as membrane-filtration processes. Nano-filtration [18,19,20] reverse osmosis [21], electro-dialysis [22,23,24] and sorption techniques [25,26,27,28,29,30] are the most conventional methods due to higher production costs and regeneration difficulties. There is a trend towards developing sustainable water treatment materials and processes [31,32] and consequently, the possibilities of the use of new synthetic material—with low cost and easy availability—are desirable to be evaluated for wastewater management.

Functionalized mesoporous materials are interesting adsorbents in comparison with other commercial DVB or styrene base adsorbents because of their attractive properties, such as a high level of heavy metal removal capacity, large surface area, narrow pore size distribution and the specificity of the chelating agents inserted in the network. An emerging group, the synthesized materials are pillared clay structures (PILCs) and similar pillared porous phosphate heterostructures (PPH) [33,34]. These materials can be specifically designed for their use for Hg(II) and Ni(II) remediation from the electrochemical industry [35], or catalytic applications [36,37,38,39,40].

We investigate herein, for the first time to the best of our knowledge, the use of PPH modified with functional phenyl groups as a new class of adsorbents for dyes from wastewater. These solids are synthesized from different combinations of silicon precursors and surfactant along with various combinations of condensation processes of alkoxides. The effect of the adsorbate was studied in order to achieve optimal conditions for the adsorption of the azo dye Acid blue (AB113), proving to be an appropriate and cost effective alternative to remove it from wastewater.

## 2. Materials and Methods

### 2.1. Synthesis of Φ_x_-PPH Adsorbent Materials

The synthesis of Φ_x_-PPH materials was carried out as described elsewhere [35] by co-condensation of phenyl-triethoxysilane (PTES) with tetraethylorthosilicate (TEOS) in the interlayer region of zirconium phosphate. Thus, cetyltrimethylammonium (CTMA)-expanded zirconium phosphate (CTMAZrP) was prepared from a solution of CTMA-Br in 1-propanol, H_3_PO_4_ (85%) and zirconium(IV) propoxide (70%) according to previously reported procedures [33]. The CTMAZrP obtained was suspended in water (10 g/L) and a solution of hexadecylamine in 1-propano (0.145 M) was added as a co-surfactant. Increasing the surfactant content in the interlayer region improves the hydrolysis and condensation of the mixture of different alkoxides. After one day of stirring, a solution of TEOS (50%, *v*/*v* in 1-propanol) and the corresponding PTES—with TEOS/PTES molar ratios of 5, 25 and 50—were added, and in each case the resulting suspension was stirred at room temperature for three days. The obtained solids were centrifuged, washed with ethanol and dried at 60 °C in air. After this stage, silica galleries are formed between the layers of zirconium phosphate and the surfactant molecules are located inside these galleries. To release this space, it has proceeded to the removal of the surfactant by cation exchange with a solution of HCl in ethanol, because the calcination process is not a suitable procedure due to the removal of the phenyl functionalized silica walls in the galleries. After various tests, both the extractor solution concentration and the number of extractions were optimzed, yielding greater efficiency in the extraction process (three times) at a concentration of HCl:ethanol (1:10; *v*:*v*). Three materials with different TEOS/PTES molar ratio added (×) are denoted as Φ_x_-PPH. All reagents used were purchased from Sigma-Aldrich (Barcelona, Spain).

### 2.2. Adsorption Experiments AB113 in Solution Data Analysis

Batch adsorption experiments were performed at room temperature adding 50 mg of Φ_x_-PPH at different volumes (a range of volumes was used) of 10^−5^ M of AB113. After one day, the solid was removed by centrifugation and the solution was analysed by UV-Vis spectroscopy. The Langmuir model assumes that the sorption sites are identical and energetically equivalent due to its homogeneous structure [41,42]. The equilibrium is obtained when the monolayer formation on the sorbent occurs. The Langmuir isotherm is described according to the following equation (Equation (1)):

(1) $$q_{e}=\;\frac{q_{m}K_{L}C_{e}}{1+\;K_{L}C_{e}}$$

where, *q_e_* (mol/kg) and *C_e_* (mol/L) are the equilibrium concentrations of AB113 in the solid and the liquid phase, respectively, *q_m_* (mmol/g) is the maximum sorption capacity and *K_L_* (L/kg) is the Langmuir constant related to the energy of adsorption. *q_e_* is obtained according to Equation (2):

(2) $$q_{e}=\left(C_{i}\;-\;C_{f}\right)\frac{V}{m}$$

where, *C_i_* and *C_f_* are the concentrations of AB113 at the beginning and the end of the adsorption process, *V* is the solution volume used during batch experiments (a range of volumes was used) and m is the mass of Φ_5_-PPH used.

The Freundlich model was used to describe the adsorption of contaminants on heterogeneous surfaces consisting of sites with different exponential distribution and energies [41,42]. The equation of the Freundlich sorption isotherm is expressed by Equation (3):

(3) $$q_{e}=\;K_{F}C_{e}^{n}$$

where, *K_F_* and *n* are the Freundlich adsorption isotherm constants, being indicative of the adsorption extension and the degree of the surface heterogeneity. The SIPS isotherm combines both Freundlich and Langmuir isotherms, which at low adsorbate concentration behaves as the Freundlich isotherm [41] and at high concentration it predicts a monolayer adsorption capacity characteristic to the Langmuir model [41]. The mathematical representation of this model is given by Equation (4):

(4) $$q_{e}=\;\frac{q_{m}{\left(K_{s}C_{e}\right)}^{n}}{1+\;{\left(K_{s}C_{e}\right)}^{n}}$$

where, *q_m_* (mol/kg) is the maximum adsorption capacity, which can also be expressed as Nt, a measure of the total number of binding sites available per g of sorbent, *K_S_* is the affinity constant for adsorption (L/kg) and *n* is the Freundlich parameter that takes into account the system heterogeneity. The SIPS isotherm is reduced to the Langmuir form for *n* = 1 and a homogeneous surface is considered. The greater the difference from this value, the greater the solid surface heterogeneity.

### 2.3. Characterization Methods

UV-Vis measurements were carried out with a Shimadzu UV-1800 spectrophotometer (Shimadzu Corporation, Kyoto, Japan). Powder diffraction patterns were collected on an X’Pert Pro MPD automated diffractometer (PANalytical, Almelo, The Netherlands) equipped with a Ge(111) primary monochromator (strictly monochromatic Cu-K_α_ radiation) and an X’Celerator detector (PANalytical, Almelo, The Netherlands). Textural parameters were obtained from N_2_ adsorption-desorption isotherms with a Micromeritics ASAP 2020 (Micromeritics Ltd., Bedfordshire, UK). The specific surface area and pore volume of the Φ_x_-PPH were determined by the adsorption-desorption of N_2_ at −196 °C. Before analysis, the Φ_x_-PPH samples were degassed at 150 °C up to 10^−4^ Torr. Pore volume and an average pore diameter were determined by the Barrett, Joyner, Halenda model. X-ray photoelectron spectra (XPS) were obtained using a Physical Electronics PHI 5700 spectrometer with a non-monochromatic Mg K_α_ radiation (300 W, 15 kV, *hν* = 1256.3 eV) as the excitation source. Spectra were recorded at 45° take-off angles by a concentric hemispherical analyser (Physical Electronics, Inc., Chanhassen, MN, USA) operating in the constant pass energy mode at 25.9 eV, using a 720 μm diameter analysis area. Under these conditions the Au 4*f*_7/2_ line was recorded with 1.16 eV FWHM at a binding energy of 84.0 eV. The spectrometer energy scale was calibrated using Cu 2*p*_3/2_, Ag 3*d*_5/2_ and Au 4*f*_7/2_ photoelectron lines at 932.7, 368.3 and 84.0 eV, respectively. Charge referencing was done against adventitious carbon (C 1*s* 284.8 eV). The C 1*s* signal of some samples was also studied with an Al K_α_ radiation due to the presence of sodium to avoid the overlapping of the Na KLL Auger signal at 290.3 eV. Solid surfaces were mounted on a sample holder without adhesive tape and kept overnight in a high vacuum in the preparation chamber before they were transferred to the analysis chamber of the spectrometer. Each region was scanned with several sweeps until a good signal-to-noise ratio was observed. The pressure in the analysis chamber was maintained lower than 10^−9^ Torr. A Shirley-type background was subtracted from the signals. Recorded spectra were always fitted using Gauss-Lorentz curves in order to determinate more accurately the binding energy of the different element core levels. The accuracy of binding energy (BE) values was within ±0.1 eV.

## 3. Results and Discussion

### 3.1. Adsorbent Characterization

Elemental analysis does not provide enough information to determine the presence of phenyl groups. The residual surfactant and the alcohol adsorbed used in the extraction procedure can interfere in the carbon content (%C). Therefore, the phenyl incorporation cannot be determined by CHN elemental analysis. However, the increasing %C when phenyl groups are grafted was observed. Thus, for the extracted materials in which the amount of surfactant present is residual, the observed differences in the %C are remarkable, being this C % higher for material Φ_5_-PPH (9.04%) and reduced for Φ_50_-PPH (2.62%), as displayed in Table 1.

**Figure 1:**
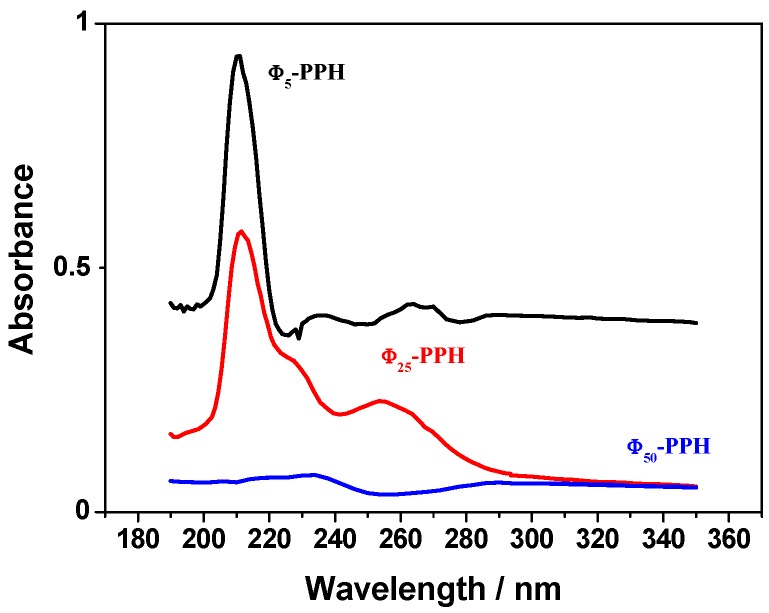
UV-Vis spectra of Φ_5_-PPH, Φ_25_-PPH and Φ_50_-PPH at 210 nm in hexane.

The concentration of phenyl groups in the synthesized material was determined by UV-VIS spectroscopy at λ = 210 nm in hexane. As seen in the UV-VIS spectra (Figure 1), the absorption due to the phenyl groups decreases with the TEOS/Φ molar ratio. A calibration curve was performed by hydrolysis of different amounts of phenyl triethoxysilane and the amount of phenyl groups incorporated in each material was determined (Table 2). As expected, the highest observed incorporation of phenyl groups occurred with sample Φ_5_-PPH, although the found value (0.047 mmol/g) was below the theoretical one if all the phenyl groups added were incorporated to the structure. This fact may be due to steric effects and to the non-polar character of phenyl groups which prevents their presence in large amounts in the interlayer space, giving a low incorporation when the silica galleries are formed upon hydrolysis and condensation of PTES, together with the TEOS.

This also justifies the found number of functional groups incorporated into the material Φ_25_-PPH that is only slightly lower than that found for Φ_5_-PPH, despite the fact that the amount of functionalized alkoxide added to the latter was five times the added amount in the case of Φ_25_-PPH. However, in spite of the increase in the number of phenyl groups incorporated, the final absorbance remained constant. Finally, as expected, in the case of material Φ_50_-PPH, the incorporation of phenyl groups in this material is very low.

After removing the surfactant used as a template, the inner silica galleries are free and a porous material is obtained. For hybrid Φ_×_-PPH, the surface phenyl groups are directed to the inner porous material. This is true if the inorganic framework is preserved after surfactant removal.

**Figure 2:**
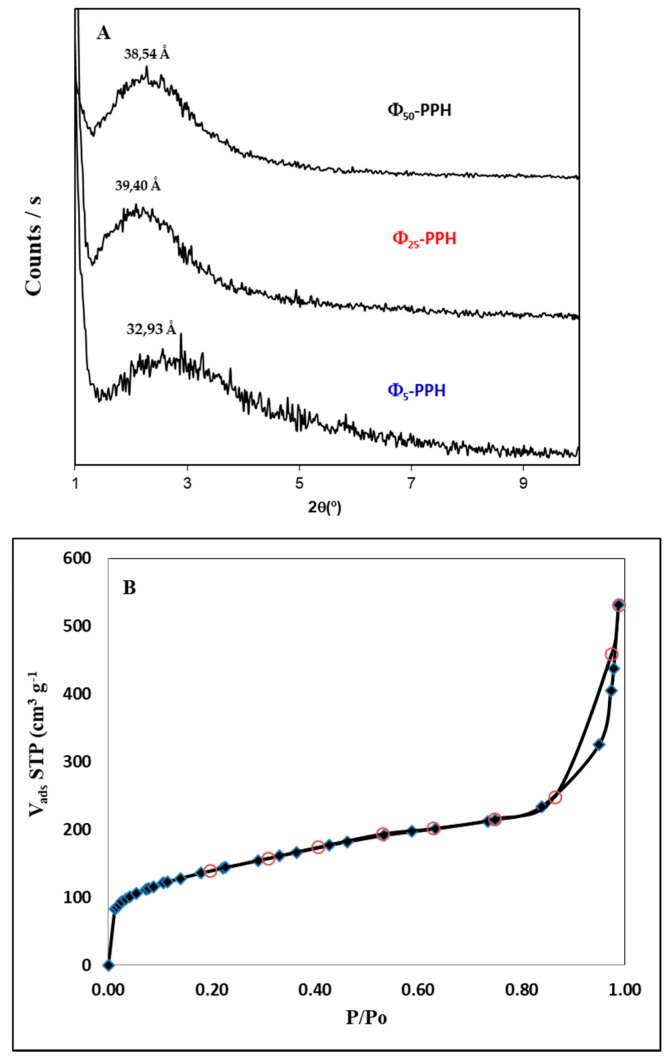
(**A**) XRD pattern of Φ_5_-PPH, Φ_25_-PPH and Φ_50_-PPH; (**B**) N_2_ adsorption-desorption isotherms at 77 K of Φ_5_-PPH.

This fact can be determined by studying the corresponding XRD diffractograms. The results show a single broad signal at a low angle corresponding to the d_001_ reflection, showing the separation of the layers of zirconium phosphate (Figure 2A). This confirms the presence of silica galleries in the interlayer region of zirconium phosphate, which keep these layers separate when the surfactant has been removed.

In Φ_5_-PPH, the diffraction peak appears at a slightly lower angle so that the basal spacing is 32.9 Å, the intensity is weak and the definition of the peak poor. This indicates that the presence of phenyl groups hinders the arrangement of the internal phase material. This low crystallinity can be justified by the steric hindrance of the phenyl groups, due to their non-polar character, since the surfactant micelles which act as structural elements of the silica galleries present a polar end placed in this environment which hinders the correct formation of the inorganic structure, mainly for the precursors which contain the phenyl group. This fact is reflected, as discussed in the previous section, in the incorporation of organic groups where the amount of phenyl groups is lower than the other functionalized PPH studied [35].

The evaluation of the typical textural parameters such as surface area and pore size distribution will confirm the formation of silica in the galleries of the interlayer region. Textural parameters obtained from the corresponding Nitrogen adsorption-desorption isotherms of N_2_ at −196 °C correspond to type IV (IUPAC) [36,37], characteristic of mesoporous materials (Figure 2B, Table 2). The surface area values (S_BET_) obtained are quite high (above 500 m^2^/g), indicating a large accessible surface within the material due to the presence of silica galleries in the interlayer region being lower than the pure heterostructures (620 m^2^/g) [33], showing that the incorporation of phenyl groups decreases the surface area of the material. Regarding the volume of pores (V_p_), these materials have very high values, even more than the pure silica heterostructures (0.543 cm^3^/g). This may be due to the presence of larger pore diameters as shown in the distribution of pores.

The surface chemical composition of the adsorbent Φ_5_-PPH before and after contact with AB113 (sample Φ_5_-PPH + AB113) is shown in Table 3. Upon contact with the dye, the percentages of C and N increase and S are now detected. However, Na was not detected indicating that the dye anion was taken up. The empiric formula of the dye anion is C_32_H_21_N_5_O_6_S_2_ and the theoretical N/S atomic ratio is 2.5. The observed N/S atomic ratio after incorporation of the dye is 2.6, a value very near the theoretical one indicating the incorporation of the dye. Sulfur is the only element of the dye that is not present in the adsorbent Φ_5_-PPH and the S 2*p* core level spectrum for sample Φ_5_-PPH + AB113 shows a S 2*p*_3/2_ contribution at 168.2 eV assigned to the presence of S(VI) as the sulfonic group of the dye [43].

### 3.2. Adsorption Isotherms

From the results obtained after chemical, structural and textural characterization, Φ_5_-PPH material was selected as adsorbent for AB113, given that it presents the highest phenyl group surface density (Table 1). Also, to evaluate the effect of the phenyl group on the AB113 adsorption, the pristine PPH material was used as a reference. AB113 adsorption isotherms were investigated in triplicate at 25 °C and pH 6.5.

**Figure 3:**
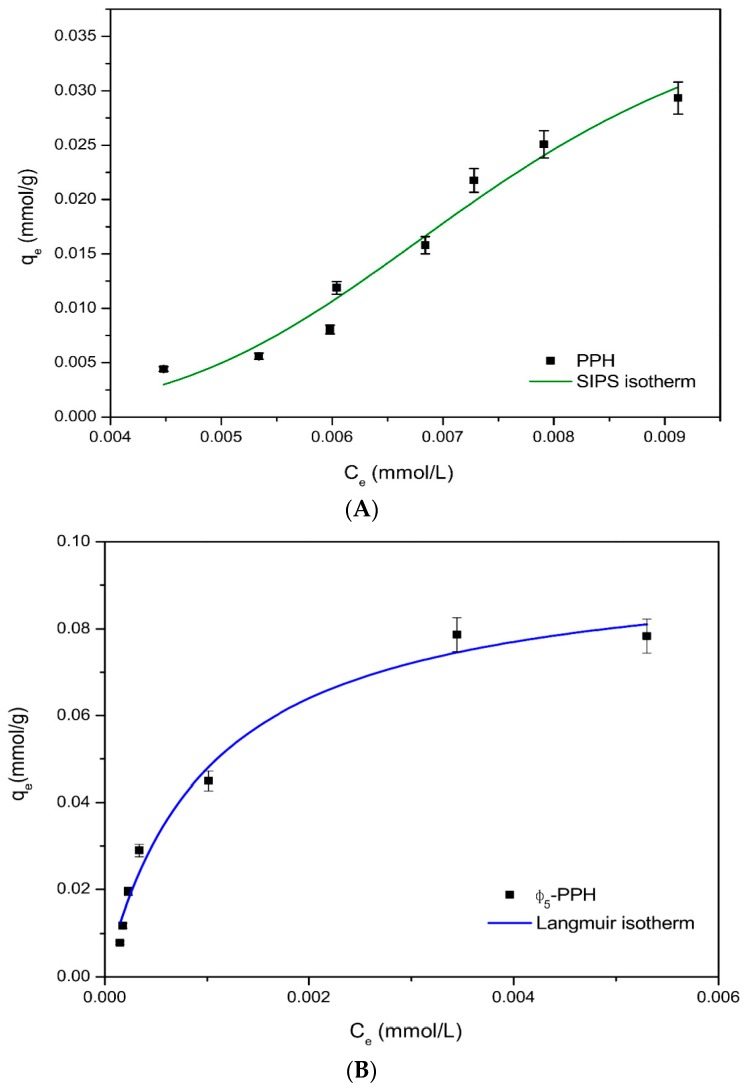
AB113 adsorption isotherms in (**A**) PPH and (**B**) Φ_5_-PPH and in relation to mmol AB113 for g of sorbent.

Figure 3 shows the fitting of the experimental data to the adsorption isotherms for PPH and Φ_5_-PPH respectively. In the case of pristine PPH, the isotherm was fitted to the SIPS isotherm with a saturated adsorption capacity of 0.04 mmol/g (Figure 3A). The adsorption data from Φ_5_-PPH were fitted to a Langmuir isotherm model, showing a saturated adsorption capacity (Q_o_) of 0.0967 mmol/g and a Langmuir constant (K_L_) of 613 L/Kg (Figure 3B). Other model fittings are shown as supplementary information as Figure S1.

The adsorption effectiveness was also evaluated. The retention effectiveness was close to 100% for Φ_5_-PPH material, when the adsorption capacity was lower than 0.040 mmol/g. From this point it decreases while increasing the amount of AB113 in the solution. For the pure PPH material, the highest retention percentage reached was 55% for the first fit, decreasing in the next points. In the literature, AB113 adsorption on mesoporous activated carbon is reported [44], where the observed adsorption capacities are in the order obtained for Φ_5_-PPH. In this case the adsorption capacities for an activated carbon obtained from a rubber tyre and for a commercial activated carbon obtained from the Langmuir isotherm (Q_o_), were 0.014 and 0.011 mmol/g, respectively, although for both activated carbons the specific surface areas are higher than that observed for Φ_5_-PPH with values of 562 and 1168 m^2^/g. Another adsorbent based in red mud obtained a Q_o_ = 0.112 mmol/g [45], demonstrating the feasibility of the proposed system to be applied.

**Figure 4:**
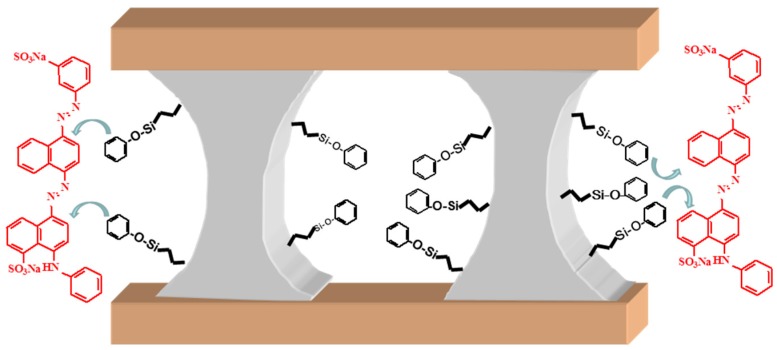
Proposed mechanism of interaction between Φ_5_-PPH and AB113.

The difference in the adsorption capacities of PPH and Φ_5_-PPH can be explained by the difference in the intermolecular forces between the different surface groups and the dye monomer. In the case of PPH, the main forces involved are strong hydrogen bonding with the AB113 (Si-OH and P-OH) and the presence of aromatic rings on the surface of phenyl-functionalized Φ_5_-PPH which offer a superior degree of delocalization due to the π-π stacking of the phenyl surface groups of the adsorbent (Figure 4). The different mechanisms of adsorption are also indicated by the fit of sorption data to two distinct isothermal models. The better fit of the Φ_5_-PPH sorption data to the Langmuir isotherm reveals that the sorption sites are identical and energetically homogeneous, suggesting that the coordination between AB113 and the aromatic rings of Φ_5_-PPH is the main mechanism of adsorption involved. However, the better fit of PPH sorption data to the SIPS isotherm indicates that there is more than one type of site involved in this process, each characterized by distinct energies and distinct affinities to adsorb. In this case, it is particularly related to the adsorption on the Si-OH and P-OH groups of PPH. To demonstrate the mechanism described, decreasing a specific area in our material does not limit increased dye adsorption.

The various applications of phenyl functionalized adsorbent have been reported in recent literature [46,47,48,49]. However, the current work is unique in terms of the use of phenyl-PPH for dye adsorption, which is reported here for the first time.

### 3.3. Application in Wastewater

**Figure 5:**
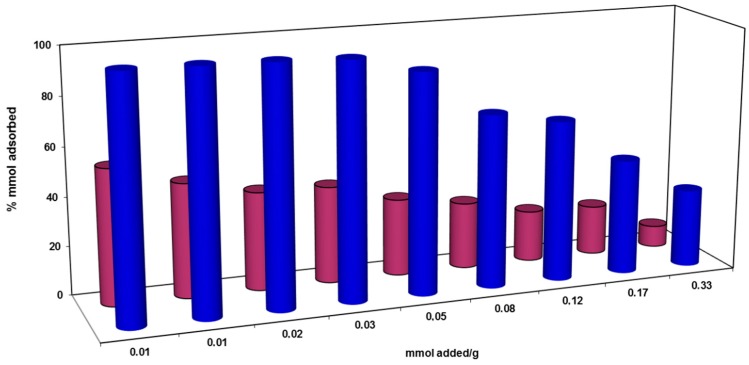
Remediation of an azo dye from industrial textile wastewater by Φ_5_-PPH recorded at 270 nm (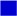) and compared with raw PPH (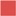).

In accordance with the results previously obtained, Φ_5_-PPH was used as an adsorbent material for treating real wastewater from a local textile stamp factory. Figure 5 shows the absorbance at 270 nm of water from a rinse step treated with different mass sorbent, where the absorbance at 270 nm achieves the minimum from a mass/volume ratio of 10 g/m^3^, which indicates that this type of hybrid phenyl material can be a promising dye scavenger.

## 4. Conclusions

The functionalized porous phosphate heterostructure materials with phenyl functionalized surface proved to be effective adsorbents for the removal of AB113 azo dye from wastewater from the textile industry. With a surface area (SBET) of 500 m^2^/g, the best known adsorption process is Langmuir, compared to the other tested models such as Freundlich and SIPS, showing saturation (Q_o_) of 0.0967 mmol/g. An adsorption mechanism is proposed between the phenyl groups of the Φ_5_-PPH and AB113, based on the affinity between both groups due to π-π stacking, suggested by the Langmuir model. The results indicate that Φ_5_-PPH is a good alternative to remove the azo dye from industrial wastewater, as shown in the application studied and supported by XPS analysis of the surface of Φ_5_-PPH after the application of the N/S ration.

## Figures and Tables

**Table 1:** Chemical composition of Φ-PPH material.

Material	%C	%N	%C *	%N *
Φ_5_-PPH	9.04	0.147	19.53	1.30
Φ_25_-PPH	3.81	0.217	18.91	1.27
Φ_50_-PPH	2.62	0.222	18.53	1.28

* Previous surfactant extraction.

**Table 2:** Characteristics of the different materials synthesized.

Material	mmol Φ/g	mmol P/g	Φ/P (Exp)	Φ/P (Exp)	S_BET_ (m^2^/g)	Vp (cm^3^/g)	C_BET_	S_ac_ (m^2^/g) *	ΣV_p_ (cm^3^/g) ^●^	Dp (Å)
Φ_5_ −PPH	0.047	1.73	0.027	0.6	498	0.516	133	482	0.472	39.13
Φ_25_– PPH	0.042	1.86	0.023	0.12	545	0.596	123	540	0.565	41.87
Φ_50_– PPH	0.003	1.78	0.002	0.06	571	0.657	133	546	0.609	44.59

* Previous tensioactive extraction and **^●^** Determinated by the Cranston e Inkley method.

**Table 3:** Atomic concentration (%) of samples Φ_5_-PPH and Φ_5_-PPH + AB113 determined by XPS.

Sample	C	N	O	Si	P	Zr	S
Φ_5_-PPH	24.50	0.50	50.07	19.55	3.52	1.86	-
Φ_5_-PPH + AB113	29.56	0.94	46.00	18.42	2.19	1.69	0.36

## Supplementary Materials

The following are available online at www.mdpi.com/1996-1944/10/10/1111/s1, Figure S1: Fitting of experimental adsorption of AB113 on Φ_5_-PPH.
