# Supplementary material for: The Application of Functionalized Pillared Porous Phosphate Heterostructures for the Removal of Textile Dyes from Wastewater
Source: Materials (Basel). 2017 Sep 21;10(10):1111. doi: 10.3390/ma10101111 (PMC5666917; doi:10.3390/ma10101111)
Supplement: Supplementary file 1 [file materials-10-01111-s001.pdf]

Supplementary Information

Application of functionalized pillared porous phosphate heterostructures for removal of textile dyes from wastewater

José Jiménez-Jiménez, Vanessa Guimarães, I. Bobos[b], Enrique Rodríguez-Castellón, Manuel Algarra

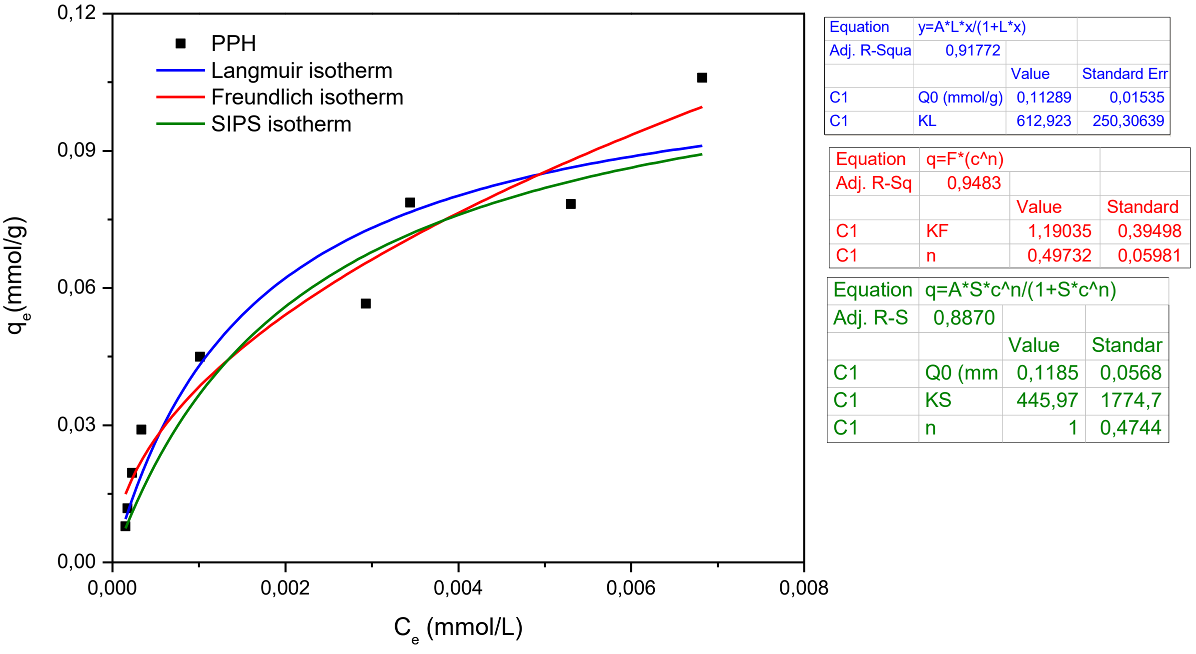

Figure SI 1. Fitting of experimental adsorption of AB113 on Φ_5-PPH
